# Disease Specific Bacterial Communities in a Coralline Algae of the Northwestern Mediterranean Sea: A Combined Culture Dependent and -Independent Approach

**DOI:** 10.3389/fmicb.2019.01850

**Published:** 2019-08-28

**Authors:** Gaëlle Quéré, Laurent Intertaglia, Claude Payri, Pierre E. Galand

**Affiliations:** ^1^Sorbonne Université, CNRS, Laboratoire d’Ecogéochimie des Environnements Benthiques (LECOB), Observatoire Océanologique de Banyuls, Banyuls-sur-Mer, France; ^2^UMR 9220 ENTROPIE, ‘Ecologie Marine Tropicale des Océans Pacifique et Indien’, IRD, CNRS, Université de La Réunion, Noumea, New Caledonia; ^3^Plateforme Bio2Mar, CNRS, Observatoire Océanologique de Banyuls, Sorbonne Université, Banyuls-sur-Mer, France

**Keywords:** crustose coralline algae, disease, bacterial communities, pathobiome, culture, NGS

## Abstract

Crustose coralline red algae (CCA) are important components of marine ecosystems thriving from tropical waters and up to the poles. They fulfill important ecological services including framework building and induction of larval settlement. Like other marine organisms, CCAs have not been spared by the increase in marine disease outbreaks. The white-band syndrome has been recently observed in corallines from the Mediterranean Sea indicating that the disease threat has extended from tropical to temperate waters. Here, we examined the microbiome and the pathobiome of healthy and diseased *Neogoniolithon brassica-florida* coralline algae in the Mediterranean Sea by combining culture-dependent and -independent approaches. The coralline white-band syndrome was associated with a distinct pathobiome compared to healthy tissues and showed similarities with the white-band syndrome described in the Caribbean Sea. A sequence related to the genus *Hoeflea*, order *Rhizobiales*, characterized the white-band disease pathobiome described by amplicon sequencing. No representative of this genus was isolated by culture. We, however, successfully isolated an abundant member of the healthy CCA microbiome, an *Alphaproteobateria* of the family *Rhodobacteraceae*. In conclusion, we did not identify a potential causative agent of the disease, but through the complementarity of culture dependent and independent approaches we characterized the healthy microbiome of the coralline and the possible opportunistic bacteria colonizing diseased tissues.

## Introduction

Crustose coralline algae (CCA, Corallinales, Rhodophyta) are important components of underwater seascapes. They act as major ecosystem bio-constructors owing to their ability to deposit calcium carbonate (Steneck, 1986). They strengthen coral reef frameworks in the tropics (Goreau, 1963; Rasser and Riegl, 2002), coralligenous structures in the Mediterranean Sea (see review Ballesteros, 2006), and build extensive maerl/rhodoliths beds from the tropics to the poles (Foster, 2001). CCAs are also beneficial to numerous other benthic organisms because they provide a suitable settlement habitat for planktonic larvae, and in particular for coral larvae (Harrington et al., 2004), and can induce their metamorphosis (Sebens, 1983; Morse et al., 1994; Heyward and Negri, 1999). Unfortunately, CCAs have not been spared by the increase in marine disease outbreaks observed in the past decades (Harvell et al., 1999; Ward and Lafferty, 2004). Diseases have increased mortality (Goreau et al., 1998) but have also been shown to impair CCA fundamental ecological services by weakening their skeleton (Quéré et al., 2015a) or reducing coral larvae settlement (Quéré and Nugues, 2015).

The Coralline White Band Syndrome (CWBS) is one of the most widespread disease affecting crustose coralline algae and yet its etiology remains largely unknown. This syndrome is characterized by the presence of a clean white band advancing on healthy tissues. The disease was first described in the Caribbean in the late 1990s (Vargas-Ángel, 2010) and was reported to be spreading extremely rapidly, which led to striking mortality in particular in *Porolithon*. In Jamaica, half the *Porolithon* population was killed within 6 months and up to three quarters of the population died at many Caribbean sites within a few months (Goreau et al., 1998). CWBS is now wide spread in the tropics with reports of the disease in the Pacific Ocean (Vargas-Ángel, 2010; Tribollet et al., 2011), in the Red Sea (Aeby et al., 2017) or in the Indian Ocean (personal observation). In 2015, observations in the Mediterranean Sea extended the distribution of this disease to temperate waters (Hereu and Kersting, 2016). Coralline white band disease occurrence has been associated with elevated seawater temperature (Quéré et al., 2015b) and its recent observation in temperate waters raises concerns for mediterranean coralligenous assemblages in the context of global warming.

Crustose coralline algae disease investigations have often focused on field surveys (Aeby et al., 2008; Vargas-Ángel, 2010; Tribollet et al., 2011; Quéré et al., 2015b), but recently, two separate studies conducted in Curaçao, Caribbean, on *Neogoniolithon mamillare*, have allowed a thorough investigation of the syndrome. A combination of field and histological analysis showed that the syndrome was likely chronic, slow-progressing and could weaken the CCA skeleton facilitating its invasion by borers (Quéré et al., 2015a, b). In addition, the sequencing of bacterial 16S rRNA genes showed a disease specific pathobiome associated with CWBS (Meistertzheim et al., 2017), but to date, there is still very little information about bacterial communities associated with CCAs.

A limited number of studies have shown that tropical CCAs are dominated by *Alphaproteobacteria* followed by *Gammaproteobacteria* (Barott et al., 2011; Webster et al., 2011, 2013a; Sneed et al., 2015). A noteworthy finding was that CCA microbiomes appeared to be species-specific with each CCA species harboring a characteristic bacterial community (Sneed et al., 2015). These communities can, however, change in response to environmental stress. In laboratory experiments, elevated sea water temperatures were for example associated with a decrease in *Alphaproteobacteria* and an increase in *Bacteroidetes* in tropical CCAs (Webster et al., 2011), and lower pH was linked to the appearance of new microbial taxa (Webster et al., 2013a). Microbial shifts have also been reported in marine sponges (Webster et al., 2008; Blanquer et al., 2016) or corals (Closek et al., 2014; Roder et al., 2014a; Ng et al., 2015) during disease outbreaks. To date, only one study has investigated the microbiome associated with CCA-diseased tissue (Meistertzheim et al., 2017). In that study, the white-patch disease of tropical CCAs was characterized by a higher abundance of a *Vibrio* sp., which could be the putative disease agent, but no clear causative agent was identified for the white-band disease. Despite these recent efforts, CWBS etiology thus remains unexplained.

The main goal of the study was to describe the microbiome and the pathobiome of a coralline algae assigned to the complex *Neogoniolithon brassica-florida* and its white band disease in the Mediterranean Sea as there is no information on CCA in temperate regions where the disease was reported only very recently. Our hypotheses are that (1) similarly to what is known from the Caribbean, the white-band disease found in temperate waters have a distinct pathobiome, (2) a causative agent could be isolated and grown in culture. To test these hypotheses we examined the microbiomes using a combined culture dependent and -independent approach, which has never been used in CCA disease investigations.

## Materials and Methods

### Samples Collection and Preparation

**FIGURE 1:**
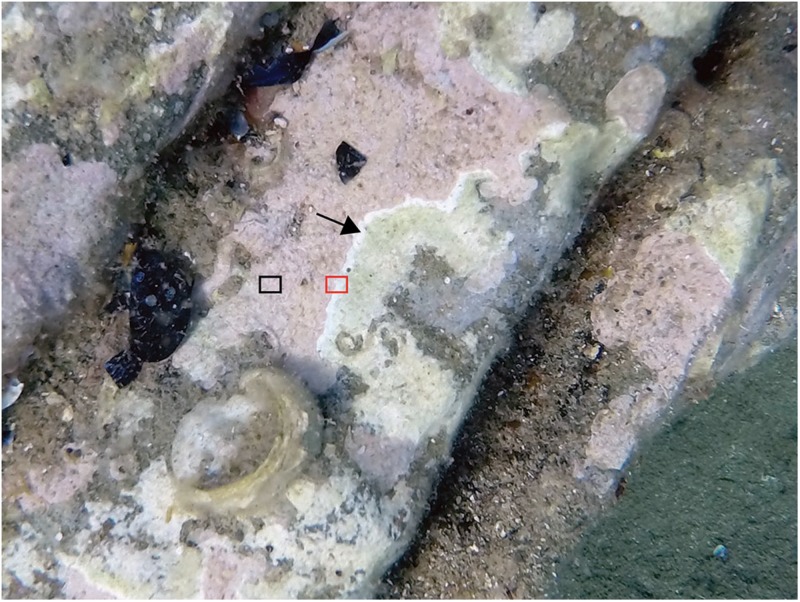
Coralline White-Band Syndrome (CWBS) in CCA, Banyuls-sur-Mer, France. Two samples were taken from each diseased CCA: healthy-looking tissue (black rectangle) and diseased tissue (red rectangle). The black arrow points to the white-band characterizing the CWBS. Additionally, samples of healthy tissues were taken from CCAs showing no visible signs of disease.

Healthy and CWBS-diseased CCA (*Neogoniolithon brassica-florida* complex) were sampled at Plage du Troc (42°N, 3°E), Banyuls-sur-Mer, France on June 15, 2017. Fragments (2–5 cm^1^) were collected using hammer and chisel at 2–4 m depth. Samples from diseased individuals included both healthy-looking and diseased tissues. Each fragment of CCA was placed in an individual collecting bag, in order to avoid contamination between specimens, and transported immediately (within 20 min) in a dark cooler to the laboratory where they were processed. Samples were taken by scratching the surface of each fragment using a sterilized scalpel blade. Each sample was fixed separately in ethanol 96% for culture-independent analysis only. A total of 15 different samples were gathered: five from healthy specimens, five from healthy-looking area of diseased individuals, and five from the boundary area between healthy and diseased looking tissues (Figure 1). CCA identification was based on morpho-anatomical observations and DNA analysis. DNA extraction was conducted under the same conditions as that used for the microbiome. Sequences were generated from the rDNAs SSU (∼1,700 bp) and the chloroplast gene psbA (∼900 bp length). See Caragnano et al. (2018) for DNA analyses conditions, sequence retrieval, alignment and phylogenetic trees.

### DNA Extraction and Amplicon Sequencing

DNA extraction and sequencing followed the protocols described in Meistertzheim et al. (2017). In short, the main steps were a mechanical lysis of the CCA sample on a FastPrep Instrument with a A Matrix (MP Biomedical, Santa Ana, CA, United States), followed by a chemical lysis by incubation with proteinase K at 57°C during 1 h and DNA extraction using the Maxwell Blood DNA Purification Kit LEV (Promega, Madison, WI, United States) on Maxwell 16 MDx Instrument (Promega). The V1–V3 region of the bacterial 16S rRNA genes was amplified using bacteria specific primers 27F (AGRGTTTGATCMTGGCTCAG) and 519R (GTNTTACNGCGGCKGCTG). All PCRs were conducted in triplicate before being pooled for sequencing. Pooled PCR products were purified using Amicon Ultra-0.5 mL Centrifugal Filters (Millipore) and sequenced at the CGEB-Integrated Microbiome Resource (IMR, cgeb-imr.ca), Dalhousie University on the same Miseq Illumina sequencer using Miseq reagent kit V3 (Illumina) producing 2 × 300-bp long reads. All sequences were deposited in GenBank under SRA accession number PRJNA524010.

### Sequence Analyses

The resulting sequence data was processed using the standard pipeline of the DADA2 package, version 1.6.0 (Callahan et al., 2016)^2^ in R 3.3.3 (R Core Team, 2017). We removed both the forward and reverse primers by cutting off the first 21 bases of all reads. After inspection of quality control profiles, the last 20 bases of all forward reads and the last 50 bases of all reverse reads were trimmed. The filtering parameters were: maxN = 0, maxEE = c(2.5), truncQ = 2. Sample sequences were then dereplicated, paired reads were merged and chimeric sequences identified and removed using the DADA2 package. DADA2 replaces the traditional “OTU-picking” step in amplicon sequencing workflows by inferring exact amplicon sequences variants (ASVs) from sequencing data. Taxonomic assignment was performed against the Silva v128 database^2^ followed by a manual BLAST search against the NCBI nucleotide collection in order to complete the taxonomic affiliation of the most abundant ASVs (Table 1).

**TABLE 1:** Most abundant ASVs obtained from NGS.

**Amplicon sequences**	**Nb of reads in healthy samples**	**Nb of reads in diseased samples**	**Order**	**Best match NCBI**	**Similarity**	**Origin**	**Reference**
ASV 434	1373	258	Rhodobacterales	MG 488668	97%	*Acropora cervicornis*	Muller et al., 2018
ASV 736	0	1399	Rhodobacterales	KY577118	97%	*Orbicella faveolata* with BBD	Muller et al., 2017
ASV 945	0	1047	Rhodobacterales	FJ403087	99%	Disease in *O. faveolata*	unpublished
ASV 1092	0	790	Rhodobacterales	JQ179010	99%	CCA under thermal stress and acidification	Webster et al., 2013b
ASV 1224	208	220	Rhodobacterales	AF365788	99%	Coral associated	unpublished
ASV 1455	479	37	Rhodobacterales	KF179676	95%	Healthy tissue of PWPS-affected *Porites*	Séré et al., 2013
ASV 1933	0	461	Rhodobacterales	FJ203262	99%	White Plague diseased corals	Sunagawa et al., 2009
ASV 1404	0	378	Rhodobacterales	KF179926	98%	*Porites* white patch syndrome	Séré et al., 2013
ASV 2046	231	56	Rhodobacterales	KF179926	99%	Diseased coral *Porites*	Séré et al., 2013

### Data Analyses

A total of 18,647 bacterial 16S rRNA gene sequences composed of 297 different ASVs were retained after removing eukaryotic sequences (chloroplast 16S rRNA from algal cells), poor quality reads and singletons. One sample from diseased CCA did not contain any sequences and was thus removed from the dataset. We found that the most appropriate way to conduct the analyses was to normalize our raw data to relative abundances (Weiss et al., 2017). The ASVs table containing reference sequences, taxonomy and proportional abundance in the different samples is available as Supplementary Table 1.

A non-metric multidimensional scaling ordination (nMDS) based on Bray-Curtis similarity was conducted to visualize similarities in community composition between samples. The nMDS was computed with the R package phyloseq (McMurdie and Holmes, 2013). Permutational multivariate analysis of variance (PERMANOVA, function adonis in the vegan package in R (Oksanen et al., 2013), and pairwise comparisons between group levels with corrections for multiple testing, function pairwise.perm.manova in the RVAideMemoire package (Hervé, 2017) were used to evaluate statistically significant differences of nMDS grouping.

To estimate alpha-diversity, the Shannon Index was calculated for each sample with the command plot_richness in the package phyloseq (McMurdie and Holmes, 2013). β-diversity was calculated between samples based on Bray-Curtis dissimilarity. It was used to compare the dispersion in bacterial community composition associated with each type of tissue. Comparison of alpha and beta-diversity values between groups was performed using a Kruskal–Wallis test and Wilcoxon pairwise tests in R.

Microbiome composition of healthy, healthy-looking and diseased tissues was characterized at the class, order, family, and ASVs levels. Significant differences in ASVs abundances between the three types of tissues were assessed using the Kruskal–Wallis test for multiple comparisons (function kruskal.pretty in R)^3^.

### Diversity of Culturable Bacteria

The CCA diseased samples that were used for the amplicon sequencing were also used for the cultivation of bacteria. Each sample was grounded using a micro-tube sample pestle in 1 ml of 0.22 μm sterilized sea water and vortexed until the sample was visually homogenous. 100 μl of the diluted (1:10, 1:100, in sterile seawater) or undiluted sample (1:1) was plated in triplicate on marine agar (MA 2216, Difco) and incubated in the dark at 22°C during 2 weeks before counting and sub-culturing. The colonies were categorized on the basis of their morphological characteristics (morphotype). Representatives of the most abundant morphotypes were further isolated for each CCA sample diluted at 1:100. Five representative colonies were picked when the morphotype had >100 colonies, three when they had > 50 colonies, 2 when > 10 colonies and 1 when < 10. The selected morphotypes were picked for two successive subculturing steps onto marine agar. Each isolate was then grown in marine broth (MB 2216, Difco) for 48 h at 22°C while being agitated (100 rpm). Each culture was cryopreserved in 5% dimethylsulfoxide or 35% glycerol, put into a −80°C freezer and added to the Banyuls Bacterial Culture Collection – BBCC^4^.

The purified strains were genotyped by partial 16S rRNA gene sequencing. Genomic DNA was extracted from a volume of 2 ml of each liquid culture. DNA extraction, PCR, and sequencing were done as previously described (Fagervold et al., 2013) using the BIO2MAR platform facilities^5^. Partial 16S rRNA gene sequences were trimmed, double checked manually, and dereplicated using the package Staden-GAP4 (Staden et al., 2003). For bacterial strain identification, each FASTA file was uploaded in Ez Biocloud (Kim et al., 2012) and compared with the cultured bacterial strain database using BLAST. Sequences from the cultured strains were also compared to ASV representative sequences by BLAST. Cultured strains sequences were deposited in GenBank under accession number: MK224651–MK224715.

Bacterial abundance (CFU mL^–1^) was determined on the basis of 1 mL measured aliquots of each sample used to prepare serial dilutions. Mean CFU counts were calculated for each health status (healthy, healthy-looking, diseased) by combining counts from all plates of each CCA replicate for a given status (*n* = 12 plate counts per health status).

## Results and Discussion

### Bacterial Community Composition

**FIGURE 2:**
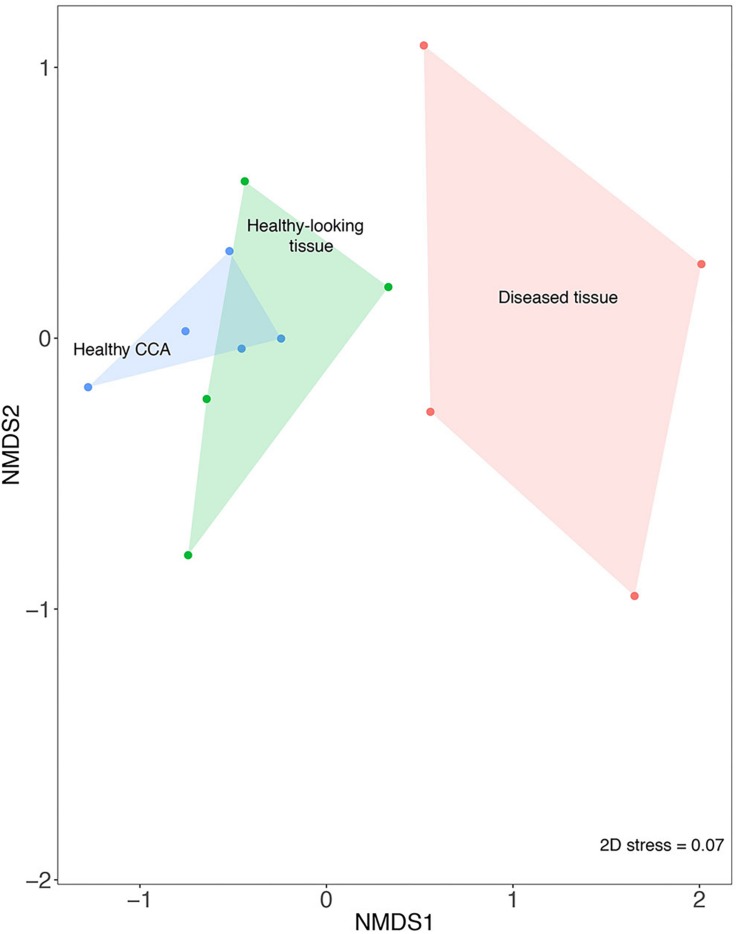
Non-metric multi-dimensional scaling plot (nMDS) based on the Bray–Curtis similarity index showing the similarity between bacterial community composition from healthy and diseased tissues in CCA affected by CWBS.

The nMDS analysis showed that samples from diseased tissues were separated from both the healthy and the healthy-looking tissue samples, which overlapped (Figure 2). The composition of the bacterial communities from the diseased tissues was significantly different from those of both the healthy and healthy-looking CCA tissues (pairwise perMANOVA, *p* < 0.01). Our results thus extend to temperate climates previous findings from the Caribbean Sea showing distinct microbiomes between healthy tissue and white-band affected tissue in *Neogoniolithon mamillare* (Meistertzheim et al., 2017). Our data strengthen the hypothesis that there is a direct correlation between the visible altered state of the CCA, i.e., the white-band lesion, and a change in its microbiome composition (Zaneveld et al., 2017).

**FIGURE 3:**
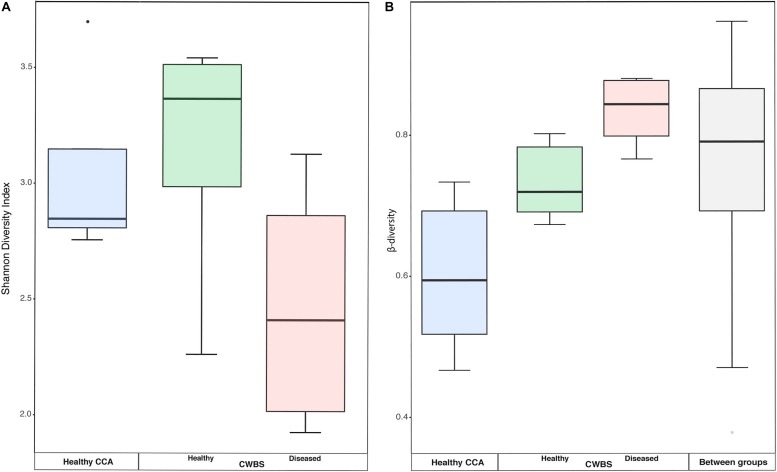
**(A)** Boxplot showing the diversity (Shannon index) based on 16S rRNA sequences from healthy and diseased CCA tissues. **(B)** Boxplots showing the beta-diversity expressed as the Bray–Curtis distance within all samples of each health group, as well as between health groups. The box represents the inter-quartile range between the upper and lower quartile. The median value is represented by the horizontal line and minimum and maximum values are represented by whiskers.

There was no significant difference in the bacterial community diversity between healthy and diseased samples as assessed by the Shannon diversity index (Kruskal–Wallis test, *p* > 0.05), but the lowest Shannon values were detected in diseased samples (Figure 3A). This result is in accordance with our earlier study that showed no difference in diversity for band disease in the Caribbean Sea (Meistertzheim et al., 2017). However, the fact that we observed the lowest Shannon values in diseased samples points toward the possibility of a reduced diversity, similarly to the one observed for another disease, the patch disease (Meistertzheim et al., 2017). Observations from other marine organisms showing increased community diversity associated to diseases (Closek et al., 2014; Roder et al., 2014a; Blanquer et al., 2016) may thus not necessarily represent the rule.

Beta-diversity values were higher in diseased samples compared to healthy samples (Figure 3B). The dispersion of beta-diversity was also higher in diseased samples (pair-wise Wilcoxon test, *p* < 0.01) (Figure 3B), which indicates that within diseased CCA tissues there was more variability in the bacterial community composition than within healthy CCA samples. We found, however, no significant differences in the homogeneity of variance between our three sampling groups. The more stable microbiomes of the healthy CCA versus the more variable diseased microbiomes may reflect a stochastic effect of the white-band disease on the bacterial community composition, rather than deterministic effects. This pattern of microbiome dynamics has been described as the Anna Karenina principle and has been observed in symbiotic communities of microorganisms responding to perturbations (Zaneveld et al., 2017). Our results show that CCA microbiomes could fulfill the same principle when the host gets sick.

**FIGURE 4:**
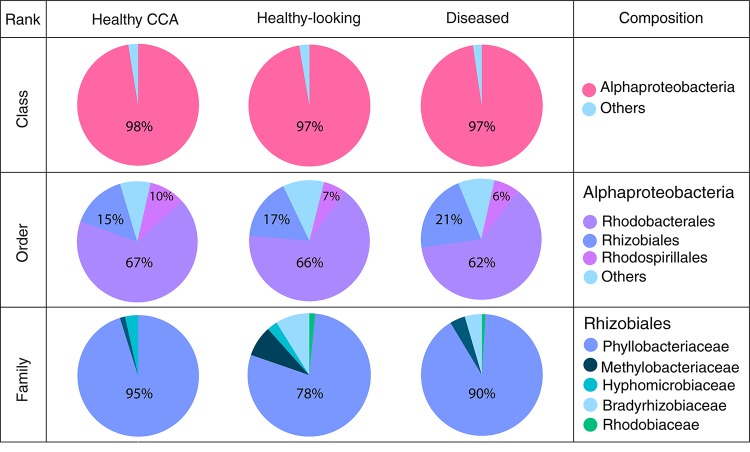
Bacterial community composition at the class, order and family levels in healthy and diseased CCA based on 16S rRNA amplicon sequences.

**FIGURE 5:**
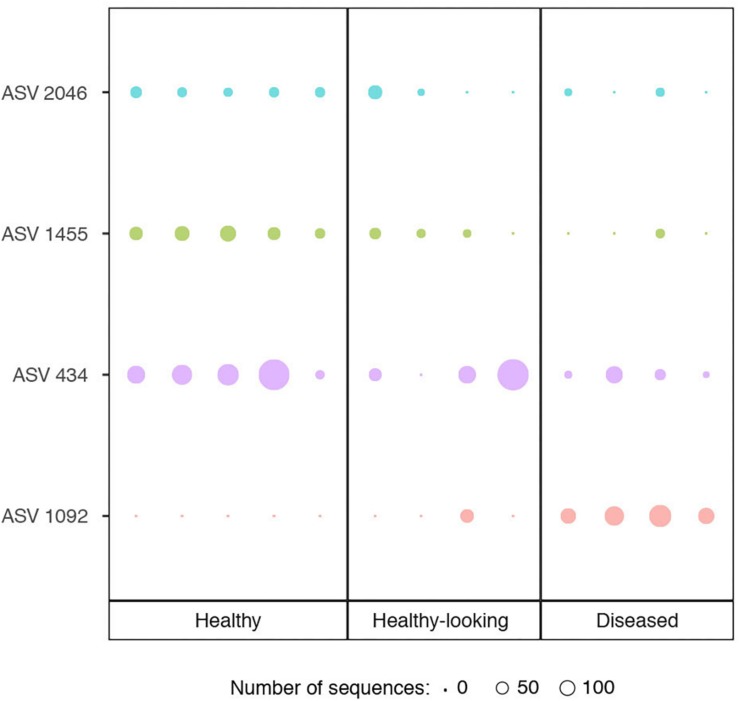
Relative abundance of ASVs (Amplicon Sequence Variants) that showed a significant difference in abundance between healthy CCA and diseased CCA (Kruskal–Wallis test, *p* < 0.05). Circle sizes are proportional to the number of sequences contained in an ASV.

Bacterial communities in each group of CCA samples were all dominated by the class *Alphaproteobacteria* (>97% of the sequences). At the class, order and family levels, there was no community difference between health status (Figure 4). In contrast, at a higher taxonomic resolution, the abundance of several ASVs significantly differed between groups (Kruskal–Wallis, *p* < 0.05) (Figure 5). White band-affected tissues were characterized by a higher proportion of the ASV 1092, which represented up to 10.7% of the sequences obtained from diseased tissues and was completely absent from healthy CCAs (Figure 5). ASV 1092 exhibited high level of similarity (99%) to a sequence assigned to the *Hoeflea* genus (*Rhizobiales*) (Supplementary Table 1), earlier described as associated with CCAs under thermal and acidification stress (Webster et al., 2013b) (Table 1). Members of the *Hoeflea* genus have been reported to be associated with skeletal growth anomalies in *Platygyra carnosus* corals or black band disease in the coral *Siderastrea sidereal* (Ng et al., 2015). In our study, this *Hoeflea* ASV was present in all four diseased samples and never detected in healthy individuals. *Hoeflea* could thus be the putative agent causing CWBS. However, it cannot be ruled out that it could be an opportunistic bacterium invading dead or dying tissues. Interestingly, the genus *Hoeflea* was not observed in CCA from Curaçao affected by the same syndrome (Meistertzheim et al., 2017). It could mean that although the syndromes observed in the Caribbean and the Mediterranean Sea are similar, they do not reflect the same disease.

Four other ASVs, ASV 736, ASV 945, ASV 1933, and ASV 1404, that all belonged to *Rhodobacterales*, were particularly abundant in diseased tissues representing up to 19, 14, 6, and 5%, respectively. They were, however, only present in one to two replicates and therefore did not show a significant difference in abundance between healthy and diseased tissue. It is nevertheless worth noting that they all had closest similarity to sequences found earlier in diseased corals (Table 1). Interestingly, ASV 1933 also showed closed similarity (99%) with one OTU (OTU 23) associated with white-band affected tissue in Caribbean CCAs (Meistertzheim et al., 2017). It indicates that they were some similarities between disease pathobiomes irrespective of location or CCA species. A consistent disease microbiome across oceans and species has recently been demonstrated for scleractinian corals that presented similar disease syndromes (Roder et al., 2014b). It remains to be demonstrated in CCAs.

In healthy CCAs, three *Rhodobacterales* ASVs, ASV 434, ASV 1455 and ASV 2046, represented up to 20, 5, and 3% of the sequences, respectively. These proportions decreased to less than 3% in diseased tissues. ASV 2046 was closely related to the *Loktanella* genus (Supplementary Table 1). Similarly, in Curaçao one of the main OTU that characterized healthy CCA samples had a 100% similarity with *Loktanella litorea* (Meistertzheim et al., 2017). In the Great Barrier Reef, a close relative of *Loktanella agnita* was found abundant in healthy CCAs. Interestingly, its abundance decreased when exposed to low pH (Webster et al., 2013b). However, *Loktanella* was not reported in the bacterial communities associated with four different CCA species in the Pacific (Sneed et al., 2015). Surprisingly, the sequenced *Loktanella* in our study was closely related to several of the bacterial strains that we isolated by culture (Table 2, 100% similarity with Culture 1 strain). Our study thus demonstrates that it was possible to isolate an important representative of the bacterial community associated with healthy CCA.

**TABLE 2:** OTUs obtained from bacterial isolates cultured from CCA samples and their best match among the ASVs obtained from NGS.

**Culture seq and matching amplicon seq**	**Nb of isolates**	**Best match Ez Biocloud**	**Similarity**	**Closest relative species**	**Family**	**Best match NCBI**	**Similarity**	**Origin or bacterial strain**	**Reference**
**Healthy**									
Culture 1/ASV 2046	6	jgi.1108058	96.61	*Loktanella sediminum*	Rhodobacteraceae	JQ179309	99%	crustose coralline algae	Webster et al., 2013b
Culture 2/ASV 1404	1	KC311338	97.10	*Loktanella sediminilitoris*	Rhodobacteraceae	KT121442	99%	*Loktanella* sp. AODO14	Saha et al., 2016
Culture 3/ASV 277	3	AY461441	99.75	*Erythrobacter aquimaris*	Erythrobacteraceae	NR_025789	99%	sea water	Yoon et al., 2004
Culture 4/ASV 29690	3	AB193438	98.82	*Tateyamaria omphalii*	Rhodobacteraceae	JQ178889	99%	CCA under therml stress and acidification	Webster et al., 2013b
Culture 5/ASV 21203	5	JAMD01000037	97.73	*Sulfitobacter pseudonitzschiae*	Rhodobacteraceae	KF799150	99%	tunicate *Ciona intestinalis*	Dishaw et al., 2014
Culture 6	2	CCJW01000022	99.80	*Vibrio crassostreae*	Vibrionaceae	KF577065	100%	coral *Oculina patagonica*	Rubio-Portillo et al., 2014
Culture 7	3	AB920327	96.89	*Spongiivirga citrea*	Flavobacteriaceae	NR_134814	97%	sponge *Tethya* sp.	Yoon et al., 2015
Culture 8	3	AJ842344	98.37	*Photobacterium rosenbergii*	Vibrionaceae	KX279509	99%	coral *Oculina patagonica*	Rubio-Portillo et al., 2017
Culture 9	1	KF740535	98.09	*Lutimonas halocynthiae*	Flavobacteriaceae	AM990866	98%	sea water	unpublished
Culture 10	3	DQ781321	98.37	*Sphingorhabdus litoris*	Sphingomonadaceae	KY787182	99%	seagrass *Posidonia oceanica*	Blanchet et al., 2017
Culture 11	2	JN578481	99.75	*Bacillus coreaensis*	Bacillaceae	KY436466	99%	mangrove plants	unpublished
Culture 12	2	CYPU01000053	99.64	*Ruegeria atlantica*	Rhodobacteraceae	KY787183	100%	seagrass *Posidonia oceanica*	Blanchet et al., 2017
Culture 13	1	GU391222	92.49	*Ferrimonas sediminum*	Ferrimonadaceae	KT731259	99%	seaweed *Delisea pulchra*	Kumar et al., 2016
**Diseased**									
Culture 14	3	CP000830	95.33	*Dinoroseobacter shibae*	Rhodobacteraceae	KF179643	98%	coral Porites	Séré et al., 2013
Culture 15/ASV 1490	3	jgi.1108064	98.61	*Loktanella maricola*	Rhodobacteraceae	AY576770	99%	*Ruegeria* sp. 3X/A02/236	Agogué et al., 2005
Culture 16/ASV 832	3	ACCU01000015	100	*Labrenzia alexandrii*	Rhodobacteraceae	FJ202588	99%	*Orbicella faveolata*	Sunagawa et al., 2009
Culture 17/ASV 3341	3	KC708867	96.96	*Pelagicola litorisediminis*	Rhodobacteraceae	AY612764	97%	Sea surface	Agogué et al., 2005
Culture 18/ASV 277	2	JMIW01000006	99.50	*Erythrobacter longus*	Sphingomonadaceae	AM691106	100%	Hypersaline spring system	Csotonyi et al., 2008
Culture 19/ASV 22655	3	jgi.1055366	97.81	*Marinovum algicola*	Rhodobacteraceae	FJ203203	98%%	*O. faveolata* diseased tissue	Sunagawa et al., 2009
Culture 20/ASV 2095	3	HG764424	92.43	*Fodinicurvata halophila*	Rhodospirillaceae	JQ179348	99%	crustose coralline algae	Webster et al., 2013b
Culture 21	3	ATUP01000002	98.99	*Oceanicaulis alexandrii*	Hyphomonadaceae	EF123309	99%	Black Band Disease coral tissues	Sekar et al., 2008
Culture 22	3	CYPU01000053	98.86	*Ruegeria atlantica*	Rhodobacteraceae	KY787183	99%	seagrass *Posidonia oceanica*	Blanchet et al., 2017
Culture 23	3	KY497472	96.44	*Alteromonas aestuariivivens*	Alteromonadaceae	KY515288	99%	marine env	unpublished
**Shared**									
Culture 24/ASV 1224	5	CP002623	97.98	*Roseobacter litoralis*	Rhodobacteraceae	KT952700	99%	surgeon fish *Acanthurus nigrofuscus*	Miyake et al., 2016
Culture 25/ASV 1933	3	jgi.1108058	97.22	*Loktanella sediminum*	Rhodobacteraceae	FJ203462	99%	coral *Orbicella faveolata*	Sunagawa et al., 2009
Culture 26	2	CYPU01000053	100	*Ruegeria atlantica*	Rhodobacteraceae	DQ888840	99%	sponge	Muscholl-Silberhorn et al., 2008
Culture 27	3	KF740534	99.87	*Ruegeria meonggei*	Rhodobacteraceae	KY787161	99%	seagrass *Posidonia oceanica*	Blanchet et al., 2017
Culture 28	3	CP013187	100	*Pseudoalteromonas phenolica*	Pseudoalteromonadaceae	GQ406775	100%	diseased gorgonian	Vizcaino et al., 2010

### Cultured Bacterial Strains

The number of bacterial colonies growing on plates (CFUs) was significantly higher in healthy and diseased CCA samples than in sea water, but there was no significant difference in abundance between healthy-looking and diseased tissues (Bonferroni–Dunn test, *p* < 0.01).

**FIGURE 6:**
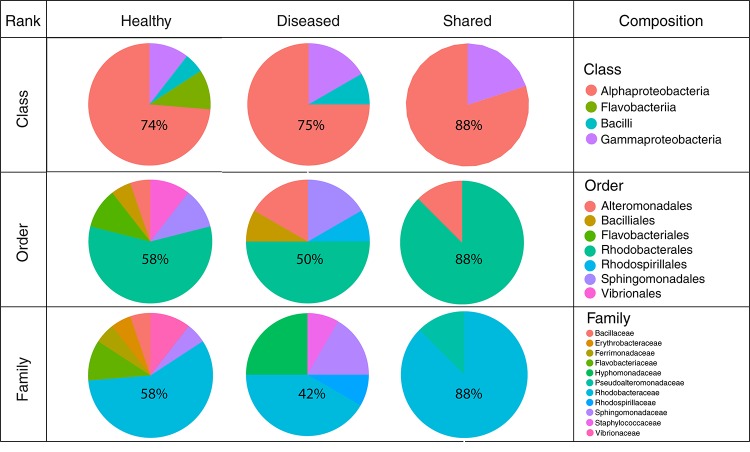
Cultured bacterial composition at the class, order, and family levels for isolates obtained from healthy-looking tissue, diseased tissue and both.

In total, 64 bacterial strains were isolated (Supplementary Table 2). After grouping strictly identical sequences, we identified 13 different isolates from healthy-looking samples exclusively, 10 from diseased samples exclusively and five that were shared among samples (Table 2). Over 70% of the cultured bacteria belonged to *Alphaproteobacteria* (Order *Rhodobacterales*, family *Rhodobacteraceae*) regardless of the type of tissue (Figure 6). Members of the *Rhodobacteraceae* (*Alphaproteobacteria*) dominated both the culturable bacterial communities and the amplicon data providing indication that they are key representatives of the CCA microbiome. *Alphaproteobacteria*, followed by *Gammaproteobacteria*, were also dominant in the bacterial communities associated with several CCA species in the Pacific (Hester et al., 2016) and CCA species in the Caribbean (Meistertzheim et al., 2017). In temperate CCAs from Tasmanian waters, *Gammaproteobacteria* were predominant in particular the genus *Moraxella* (Lewis et al., 1985).

A higher number of different classes, families and orders were represented in bacteria cultured from healthy-looking samples in comparison to diseased samples. *Gamma-proteobacteria* and *Flavobacteriia* were present in the same proportions in healthy-looking tissues (10%) followed by *Bacilli* (6%) (Figure 6). In diseased samples, *Flavobacteriia* were absent. *Gammaproteobacteria* were the second most abundant class represented (17%) followed by *Bacilli* (8%). *Vibrionales* and *Flavobacteriales* were associated with healthy-looking samples only, whilst *Rhodospirillales* were found in diseased CCA only (Figure 6).

**FIGURE 7:**
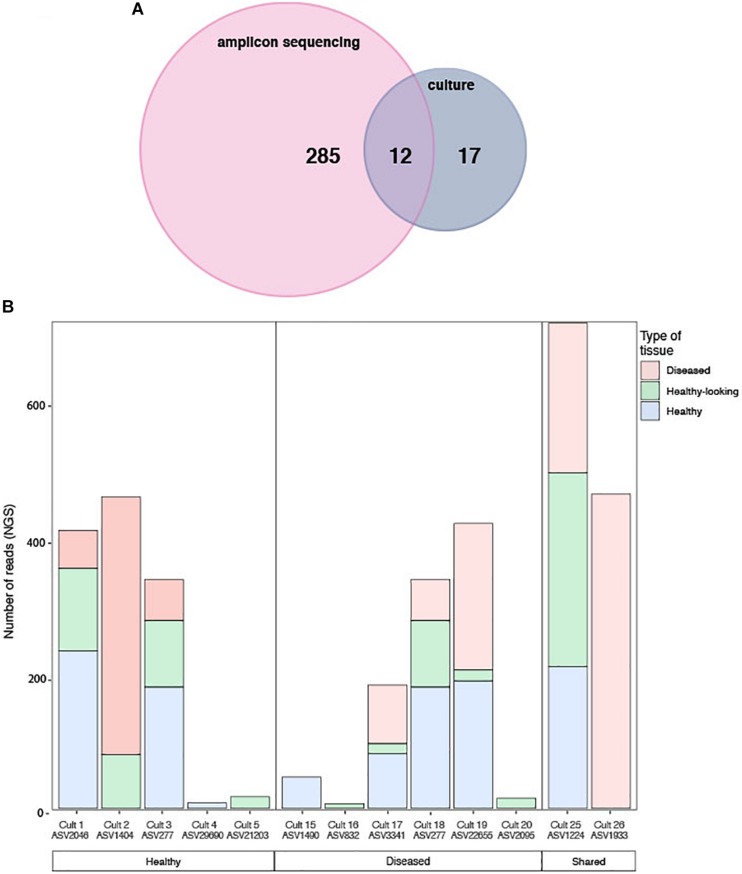
**(A)** Venn diagram of shared sequences between the ASVs obtained through amplicon sequencing and the cultured strains. **(B)** Number of sequences obtained by amplicon sequencing in healthy and diseased CCA samples for each of the 12 matching bacterial strain isolates exclusively in each health groups. Both the culture name and its ASVs equivalent are indicated.

The cultured and amplicon datasets shared 12 sequences (>99% similarity) that represented 4% of the total number of ASV, which in turn represented 18% of all the amplicon sequences (Figure 7A and Table 2). Four of those ASVs were within the 12th most abundant ASVs (>300 reads in total). We could, however, not match health group specific cultured strains with group specific amplicon sequences (Figure 7B). ASVs that had a culturable representative were not significantly more represented in one of the CCA health group compared to the others. Some sequences that were obtained in culture from diseased samples only (e.g., Culture 19 in Figure 7B) were retrieved by amplicon sequencing in similar proportions from both healthy and diseased samples. Among the bacterial strain isolates, 17 did not have a match in the ASVs obtained through amplicon sequences. The same type of discrepancies between culture-dependent and -independent approaches have been previously documented in plants where isolated strains were not found in the amplicon dataset (Dissanayake et al., 2018) or in corals with no overlap between the cultured strains and the uncultured sequences (Rohwer et al., 2001). In the culture-dependent approach, the use of a solid media may enhance the growth of certain bacteria such as those with fast growing ability (Dissanayake et al., 2018) even if they are not abundant in the tissues. This would result in a high number of isolates but no detection in next generation sequencing. However, in our study, the three most abundant cultured morphotypes had a match in the amplicon dataset.

In summary, the coralline white-band syndrome observed in temperate waters had a distinct pathobiome compared to healthy tissues. It also shared significant similarities in diversity with the white-band syndrome described in the Caribbean. Through amplicon sequencing we detected an ASV that was typical for diseased tissue, but unfortunately, this bacterium was not present within the cultured strains. It thus remains to be tested whether it is a possible causative agent of the disease. However, among the cultured strains that we isolated, we repeatedly detected a strain of *Alphaproteobateria* (family *Rhodobacteraceae*) that was also dominant within the amplicon data. In our opinion, we successfully isolated an important member of the healthy CCA microbiome that could be further analyzed by genome sequencing. The culture dependant and independent approaches were complementary, they gave similar results at the broad phylum level but showed clear differences at the finer taxonomic resolution.

## Data Availability

The datasets generated for this study can be accessed from GenBank, PRJNA524010.

## Author Contributions

GQ, PG, and LI conceived and designed the experiments. PG and CP contributed to the reagents, materials, and analyses tools. LI performed the culture experiments. All authors analyzed the data, wrote the manuscript, contributed critically to the drafts, and gave final approval for the manuscript.

## Conflict of Interest Statement

The authors declare that the research was conducted in the absence of any commercial or financial relationships that could be construed as a potential conflict of interest.

## Footnotes

1 https://www.arb-silva.de/documentation/release-128/

2 https://github.com/benjjneb/dada2

3 https://doi.org/10.5281/zenodo.1458243

4 http://collection.obs-banyuls.fr/

5 http://bio2mar.obs-banyuls.fr/en/index.html
